# Characteristics of Nasal-Associated Lymphoid Tissue (NALT) and Nasal Absorption Capacity in Chicken

**DOI:** 10.1371/journal.pone.0084097

**Published:** 2013-12-31

**Authors:** Haihong Kang, Mengfei Yan, Qinghua Yu, Qian Yang

**Affiliations:** College of Veterinary Medicine, Nanjing Agricultural University, Nanjing, People's Republic of China; The Ohio State University, United States of America

## Abstract

As the main mucosal immune inductive site of nasal cavity, nasal-associated lymphoid tissue (NALT) plays an important role in both antigen recognition and immune activation after intranasal immunization. However, the efficiency of intranasal vaccines is commonly restricted by the insufficient intake of antigen by the nasal mucosa, resulting from the nasal mucosal barrier and the nasal mucociliary clearance. The distribution of NALT and the characteristic of nasal cavity have already been described in humans and many laboratory rodents, while data about poultry are scarce. For this purpose, histological sections of the chicken nasal cavities were used to examine the anatomical structure and histological characteristics of nasal cavity. Besides, the absorptive capacity of chicken nasal mucosa was also studied using the materials with different particle size. Results showed that the NALT of chicken was located on the bottom of nasal septum and both sides of choanal cleft, which mainly consisted of second lymphoid follicle. A large number of lymphocytes were distributed under the mucosal epithelium of inferior nasal meatus. In addition, there were also diffuse lymphoid tissues located under the epithelium of the concha nasalis media and the walls of nasal cavity. The results of absorption experiment showed that the chicken nasal mucosa was capable to absorb trypan blue, OVA, and fluorescent latex particles. Inactivated avian influenza virus (IAIV) could be taken up by chicken nasal mucosa except for the stratified squamous epithelium sites located on the forepart of nasal cavity. The intake of IAIV by NALT was greater than that of the nasal mucosa covering on non-lymphoid tissue, which could be further enhanced after intranasal inoculation combined with sodium cholate or CpG DNA. The study on NALT and nasal absorptive capacity will be benefit for further understanding of immune mechanisms after nasal vaccination and development of nasal vaccines for poultry.

## Introduction

Mucosal vaccination is a promising alternative to parenteral vaccination, as it is noninvasive and, in principle, capable of eliciting strong local and systemic immune responses in mucosal-associated lymphoid tissue (MALT). Among the mucosal administration sites, nasal cavity may be the most attractive one. The highly vascularized epithelial layer and the large surface area of the nasal cavity offer great opportunities for vaccine delivery.

The nasal mucosa is the main entering site of various pathogenic microorganism [1]. Nasal-associated lymphoid tissues (NALT) are considered as the main inductive sites for immune responses in both natural infection and vaccination [2], [3]. NALT has been found in rat, mouse [4], hamster, and primates, which is considered as equivalent to Waldeyer's ring in humans [1], [5]. Most of the studies about NALT have been performed on rodents. Currently, some progress has been made in the cell types and composition of NALT in birds. Lymphoid nodules were the major NALT structures in chickens. They were composed of B cells with frequently developed germinal centers (GC), surrounded by a coat of CD4^+^ cells, while CD8^+^ cells were located in the epithelium and in the lamina propria of the nasal cavity mucosa [6], [7]. Bronchus-associated lymphoid tissue (BALT), constitutively present in normal chicken lungs [8], has the similar structures with NALT. GC was developed in most mature BALT nodules. CD4^+^ cells surrounded GC, while CD8^+^ lymphocytes were dispersed among lymphoid nodules and in the epithelium, and they rarely occurred in GC [9]. And its structures showed features characteristic for mucosal inductive sites [2], [10]. Research indicated that there were quite differences in intranasal immunization of different species, mainly due to the anatomical and histological features of nasal cavity [11]. However, the location and histological structure of NALT in chicken were not investigated systematically and detailedly.

Mucosal immunization by intranasal delivery with inactivated virus is often insufficiently effective. However, it would be highly desirable in view of the high variability of the virus and suitable mucosal adjuvants are being sought to increase its efficiency [12]. In general, the short residence time and low absorption efficiency of the vaccines restrict the application of intranasal immunization. Therefore, bioadhesive agents, absorption enhancers and immunostimulants were used to combine with inactivated virus for overcoming these bottlenecks. The studies on intranasal immunity usually focus on the effects of the immune responses and the rate of protection by means of optimizing the antigens and the adjuvants. However, it was little concerned whether the antigen could pass through the nasal epithelium or get in touch with the lymphatic tissues.

Hence, the systematic anatomical and histological characteristics of chicken NALT was determined. Then different size particles and inactivated avian influenza virus (IAIV) were intranasal inoculation to evaluate the absorbing capacity of the nasal mucosa. This study aimed at determining the absorbing capacity and position of nasal mucosa against different classified materials, which would be benefit for further understanding of immune mechanism after nasal vaccination and the development of effective nasal vaccines for birds.

## Materials and Methods

### Animals and absorbing particles

A total of 185 one-day-old White Leghorn chickens were obtained from veterinary station of Liuhe District (Nanjing, China) and housed in a controlled environment with a 12 h light-dark cycle. Food and water were provided *ad libitu*m. To evaluate the absorptive capacity of the nasal cavity, particles with different molecular weight, diameter and immunogenicity were used for intranasal inoculation, which included Trypan blue (302643, Sigma), Ovalbumin (OVA, A2512, Sigma), fluorescent latex particles (FLP) (L5155, L0780, L9902, Sigma) and Dlight 488 labeled IAIV. Trypan blue and OVA was dissolved in PBS (0.1 M, PH 7.4) for intranasal inoculation. IAIV (H9N2, A/Duck/NanJing/01/1999) was generously provided by Jiangsu Academy of Agricultural Sciences (Nanjing, China). The virus was purified by using a discontinuous sucrose density gradient centrifugation, as previously described [13]. HA protein was approximately 35% of the viral protein, so the purified virus was measured by Micro BCA Protein Assay Kit (PIERCE) and showed with the concentration of HA protein. Then the virus was labeled with the fluorescent probe DyLight 488 (Thermo Fisher Scientific) according to the manufacturer instructions, and the unincorporated dye was removed by the fluorescent dye removal columns (Thermo Scientific). The characteristics of particles are shown in Table 1.

**Table 1:** Experiment design and the characteristic of the absorption materials.

Group	Absorbing Particles	MW	Particle size	Color	Concentration	Dose
1	Trypan blue	960.82	ND	Blue	10 mg/ml	200 µl
2	OVA	44287	ND	Colorless	10 mg/ml	200 µl
3	FLP*	ND	30 nm	Fluorescent yellow-green	25 mg/ml	200 µl
4	FLP	ND	50 nm	Fluorescent blue	25 mg/ml	200 µl
5	FLP	ND	100 nm	Fluorescent red	25 mg/ml	200 µl
6	Dylight 488 labeled IAIV	ND	100–200 nm	Fluorescent green	10 HA/ml	200 µl

*Fluorescent latex particles

### Ethics Statement

All animal care and procedures were in accordance with national and institutional policies for animal health and well-being. All samples collection and study were approved by guide for care and use of laboratory animals of Nanjing Agriculture University (Nanjing, China) and the Jiangsu Provincial Academy of Agricultural Sciences. The license number was SCXK (Su) 2002–0029.

### Anatomy and histological analysis

A total of 20 chickens were euthanized by intravenous injection of pentobarbital on day 7, 21, 35 and 56 after birth, 5 chickens each time. After death, the head of the chicken was cut off along the line ventral to the upper jaw and rostral to the orbital cavity. Then, the beak in front of the nostrils was removed, and the skin was taken off. After removing the cheek muscles and muscles ventral to the orbital cavity, the remaining nose, which contained the nasal turbinates, septum, lateral walls and maxilla, was fixed in Bouin's fluid for 72 hours at room temperature [14].

2 chickens were used for the anatomical analysis each time. The blocks of noses were cut consecutively by a scalpel, in parallel with the vertical section and cross-section. The anatomical structure of the nasal cavity was observed under a stereo zoom microscope (MOTIC, SMZ-168). The remaining 3 chickens were used for histological analysis. The blocks were decalcified by 10% methanoic acid for 1 week at room temperature. After decalcifying, the blocks were embedded in paraffin and serially cut into 4 µm sections at an interval of 0.3 mm using a microtome (LEICA RM 2015). All of the sections were mounted on slides and stained with hematoxylin-eosin. Five corss-sections with the same interval were selected, and the integral images were scanned by Olympus BX51&DP 70 Digital Camera System.

### Absorption experiment and sample

90 chickens were divided into 6 groups averagely and received different particles for each group (Table 1). The absorption particles were dropped into the nostrils (100 µl/nostril) of 14-day-old chickens. Another 15 chickens was administrated 0.1 M PBS of equal doses intranasally as a control. Then 6 chickens of each group were anesthetized and sacrificed at 1 and 2 hour-post inoculation (h.p.i.) each time. The noses were sampled as described above and fixed in paraformaldehyde for 12 hours.

To evaluate whether the absorption of IAIV could be improved by the adjuvants, 60 chickens were used and divided into 4 groups averagely. Sodium cholate (C1254, Sigma) acted as an absorption enhancer and CpG DNA acted as an immunostimulant. They were intranasal inoculation combined with IAIV that have labeled by DyLight 488. Then 5 chickens of each group were killed at 0.5, 1 and 2 h.p.i. each time. CpG DNA used in our study was the bacterial genomic DNA extracted from the *E.coli Poephogus grunniens* strain. A genomic DNA extraction kit (QIAGEN) was used in this process. The doses were 50 µg CpG DNA (2.5 µl) and 1 mg sodium cholate per chicken.

### Histochemistry and immunohistochemistry for Trypan blue and OVA positive cells

The fixed tissues were embedded in paraffin and cut into 4 µm sections. Trypan blue positive cells were detected by HE staining [15]. OVA positive cells were detected by immunohistochemistry using Avidin-Biotin complex (ABC) method. The primary antibody was rabbit anti-OVA polyclonal antibody (clone OVA-14, Sigma), and the subsequent detection was completed using an ABC kit (BOSTER, Wuhan, China). The primary antibodies were replaced by common rabbit serum for negative control.

### Apoptosis assays for Trypan blue and OVA positive cells

Trypan blue is a vital stain used to selectively color dead tissues or cells blue, and generally considered can not be taken up by the living cells with intact cell membranes. To determine whether the apoptotic cells in the nasal mucosa absorbed trypan blue, nasal cavity was taken to make paraffin section, and the apoptosis of nasal tissue were detected by TUNEL assays using a cell apoptosis detection kit (BOSTER, Wuhan, China). The steps were as previously described [16]. Differently, slides in this study were colored with a DAB reagent kit bought from NanJing SunShine Biotechnology Co., LTD. (Nanjing, China). The TUNEL positive cells showed orange-brown nuclei under light microscope (Olympus BH2, Japan).

### LSCM for FLP and IAIV

The fixed tissues were embedded in OCT (Tissue Freezing Medium, SAKURA) and cut into 8 µm sections using a freezing microtome. For the detection of FLP with blue fluorescence, the sections were stained with FITC-phalloidin (Invitrogen). For the detection of FLP with yellow-green and red fluorescence and DyLight-488 labeled IAIV, the sections were stained with 4′,6-diamidino-2-phenylindole (DAPI, Invitrogen). Then the sections were observed using a Laser scanning confocal microscopy (LSCM, ZEISS LSM 710).

### Statistical analysis

The area of DyLight-488 labeled IAIV in NALT was determined per microscope field using an image analysis system (VIDS Synoptics Version 4.5.0.29, Cambridge, UK). The relative area occupied by DyLight-488 labeled IAIV positive cells was recorded. Five sections per chicken (five chickens per group) and five microscope fields per section were selected for statistical analysis. Statistical analysis was performed using Statistical Program for Social Sciences (SPSS) 16.0. The significance of the data was determined by Analysis of Variance (ANOVA). P values less than 0.05 were considered statistically significant.

## Results

### Anatomical structure of the nasal cavity

**Figure 1:**
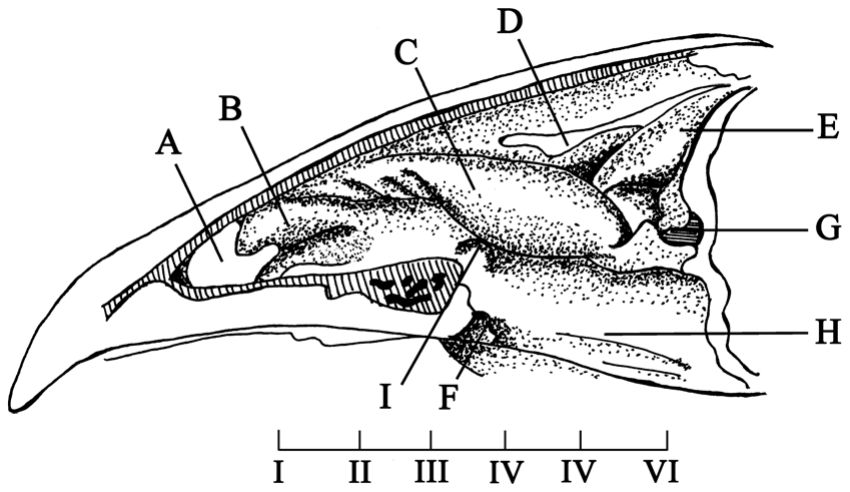
Anatomical structure of the nasal cavity through a vertical sectional view of a chicken's skull. (A) nostril, (B) concha nasalis rostralis, (C) concha nasalis media, (D) concha nasalis media, (E) concha nasalis caudalis, (F) vomer, (G) openng into sinus cavity, (H) choanal cleft, (I) opening of ductus nasolacrimalis.

**Figure 2:**
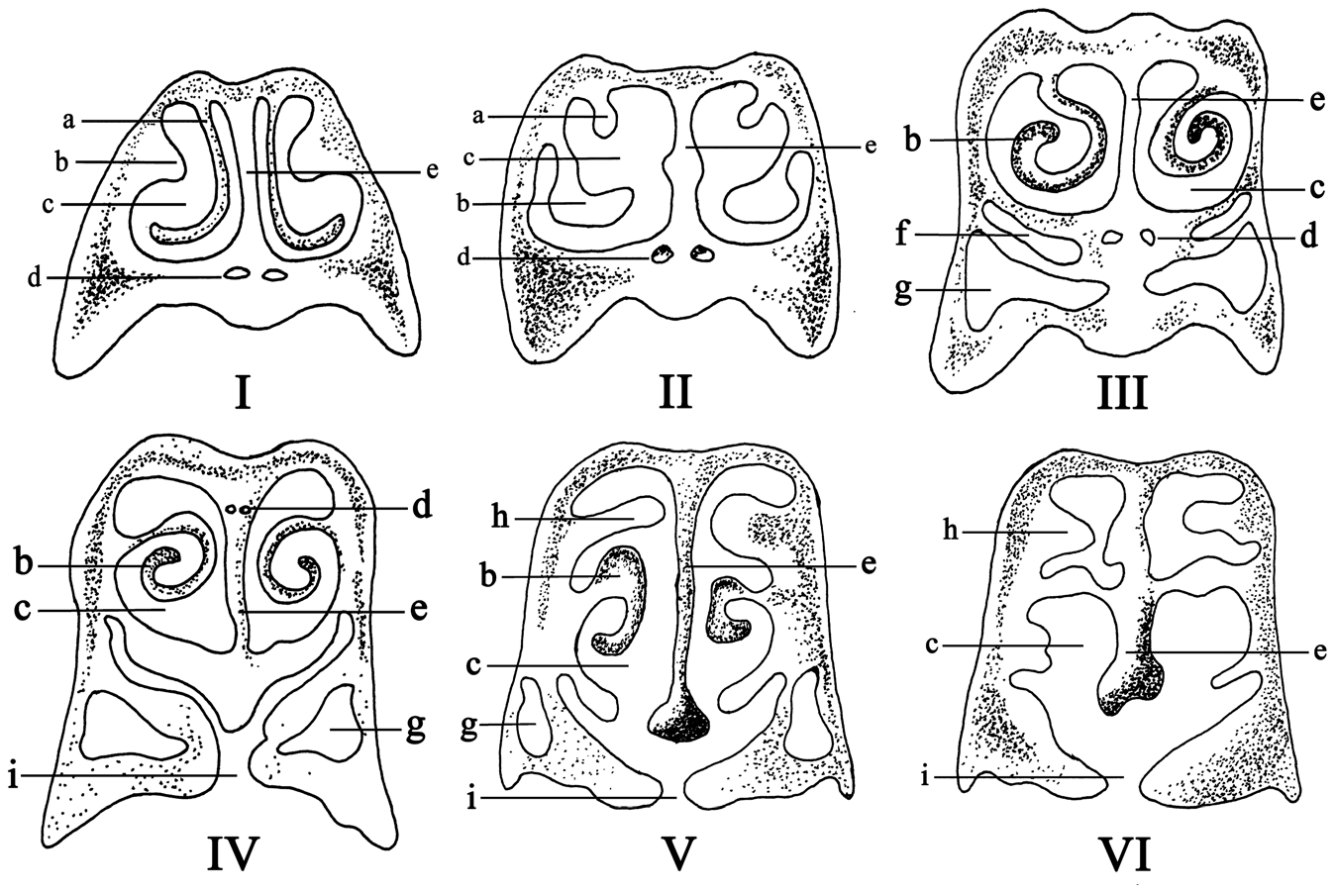
Transverse views of the six cross-sections (I–VI) from chicken's nasal cavity. (a) concha nasalis rostralis, (b) concha nasalis media, (c) nasal meatus, (d) maxillary nerve of nervi trigeminus, (e) nasal septum, (f) inferior nasal meatus, (g) infraorbital sinus, (h) concha nasalis caudalis, (i) choanal cleft.

The anatomical structure of the chicken's nasal cavity was shown in Figure 1. Six cross-sections with same interval were then selected to show the details (Fig. 2). The nasal cavity of the chicken originated from the end of the beak and ended in front of the eyepit. The dorsal part of nasal cavity was os nasale, and the ventral part was maxilla. The nasal cavity was cone-shaped and separated into right and left sides by a cartilaginiform nasal septum (Fig. 2e). The bracket of the nasal abdominal walls was composed of the palatine process of the maxilla and frontal bone, while the posterior part of the nasal cavity was mainly made up of parenchyma and was underpropped by the palatine bone and the vomer. According to the anatomy structure and epithelial tissues, the nasal cavity can be separated into three segments: regio vestibularis (RV), regio respiratoria (RR) and regio olfactoria (RO). In each part, a pair of turbinates was located on the wall of nasal cavity which termed concha nasalis rostralis (anterior turbinate, Fig. 1B and Fig. 2a), concha nasalis media (middle turbinate, Fig. 1C and Fig. 2b) and concha nasalis caudalis (posterior turbinate, Fig. 1E and Fig. 2h).

### Histological characteristics of the nasal cavity

**Figure 3:**
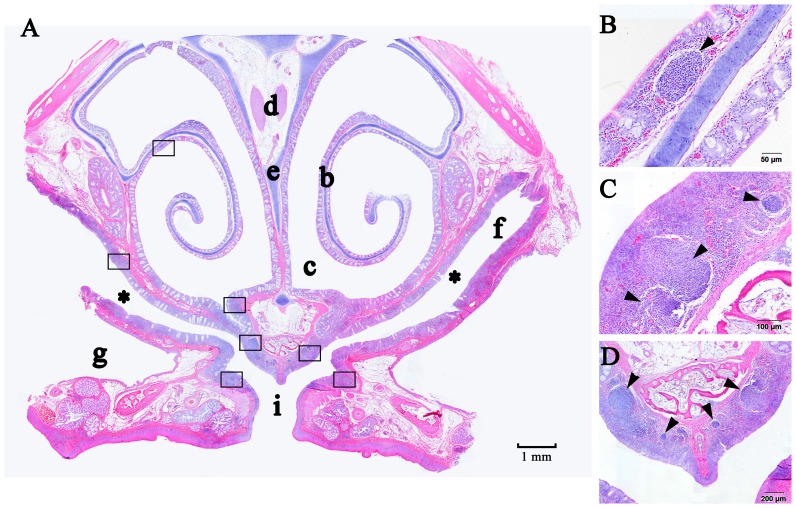
HE staining of the cross-section IV from the chicken's nasal cavity. (A) Panoramic scanning of the section, (b) concha nasalis media, (c) meatus nasi, (d) optic nerve of nervi trigeminus, (e) nasal septum, (f) inferior nasal meatus, (g) infraorbital sinus, (i) choanal cleft. (B) Diffuse lymphoid follicle covered by FAE located on the concha nasalis media. (C) NALT located on the dorsal side of choanal cleft. (D) NALT located on the nasal septum.

In the forepart of the nasal cavity (cross-section I), the epithelium was stratified squamous epithelium. Then the transfer from stratified squamous epithelium to pseudostratified columnar ciliated epithelium could be observed (cross-section II and III). In the cross-section IV, the epithelium was mainly pseudostratified epithelium consisting of ciliated cells and goblet cells. A large number of lymphoid tissues were observed in this cross-section (Fig. 3, shown with the black boxes). These lymphoid tissues were mainly secondary lymphoid follicles located in the lamina propria near the choanal cleft (Fig. 3D) and under the epithelium of walls of the inferior nasal meatus (Fig. 3A, shown with asterisk), which termed nasal-associated lymphoid tissues (NALTs). The follicle-associated epithelium (FAE) of NALT was mainly no-ciliated cells. There were still a few diffused lymphatic follicles located under the epithelium of the concha nasalis media (Fig. 3B). NALT of chicken was still visible in the cross-section V and VI, with the similar location as the cross-section IV.

### Absorption of Trypan blue

**Figure 4:**
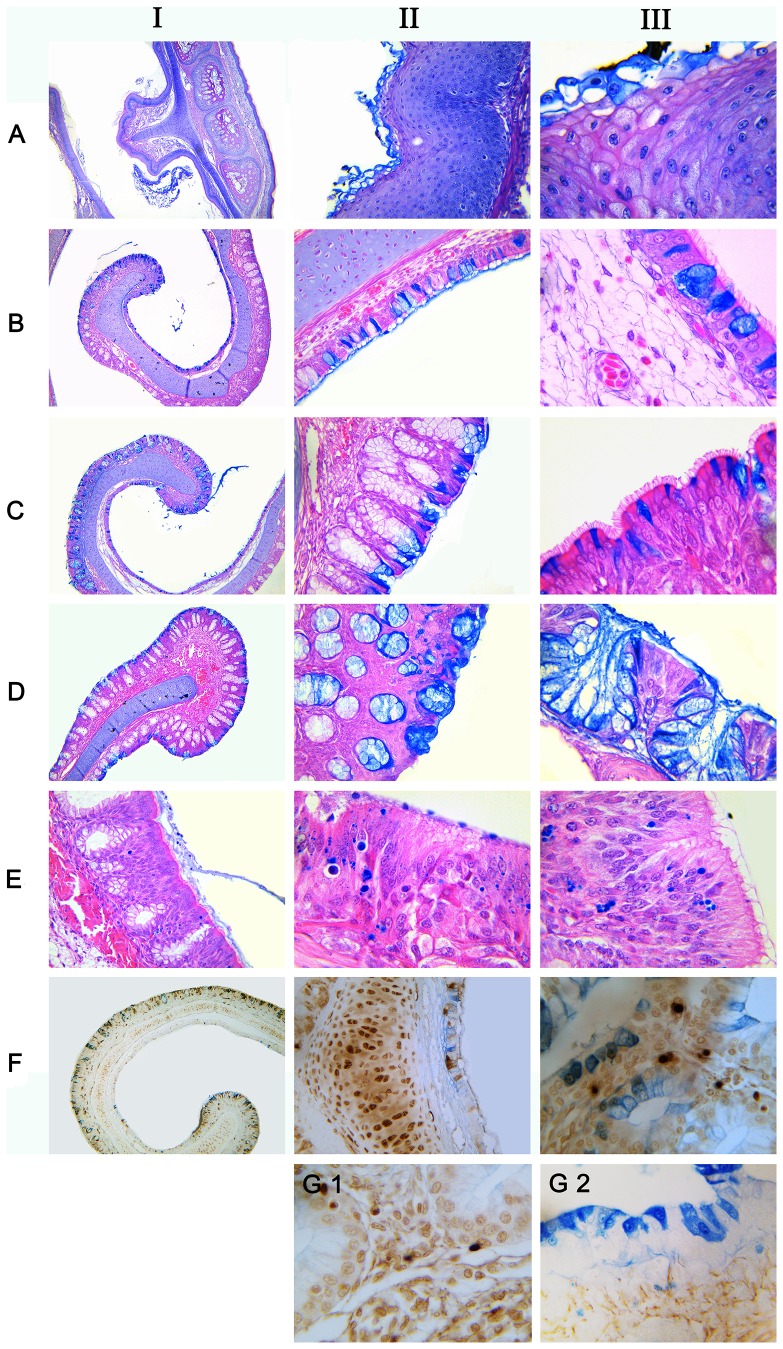
Absorption of typan blue by chicken nasal mucosa. (A) The forepart of the nasal cavity, (B) Medial side of the concha nasalis media, (C) Lateral side of the concha nasalis media, (D) Mucous glands, (E) Posterior region of the nasal cavity, (F) Apoptosis detection by immunohistochemistry on the trypan blue positive tissues. Column I×100;Column II×200;Column III×400. (G1) control (no trypan blue) ×400, (G2) iso-type control ×400. Essentially similar results were obtained in three independent experiments.

As the Figure 4 showed, trypan blue could not be absorbed by the stratified squamous epithelium in the front of the nasal cavity (Fig. 4A). On the contrary, trypan blue could enter the mucosal epithelial cells of the medial and lateral sides of the middle turbinate, including ciliated columnar epithelium, non-ciliated epithelial cells and goblet cells. After uptaking trypan blue, these cells showed dark blue (Fig. 4B and 4C). However, not all of the epithelial cells could uptake trypan blue and there was significant difference between adjacent epithelial cells (Fig. 4B and 4C). In the posterior of the nasal cavity, trypan blue could be transported to the lamina propria, far away from the mucosal epithelium, and was showed as round dot particle in the cytoplasm (Fig. 4E). However, there were not concentrated trypan blue in NALT and diffuse mucosal lymphoid tissues. It is generally believed that trypan blue could be excluded by living-cells in the cellular activity assay [17], [18]. Nevertheless, the apoptosis assays showed the cells which contained Trypan blue were not apoptotic cells (Fig. 4F).

### Absorption of OVA

**Figure 5:**
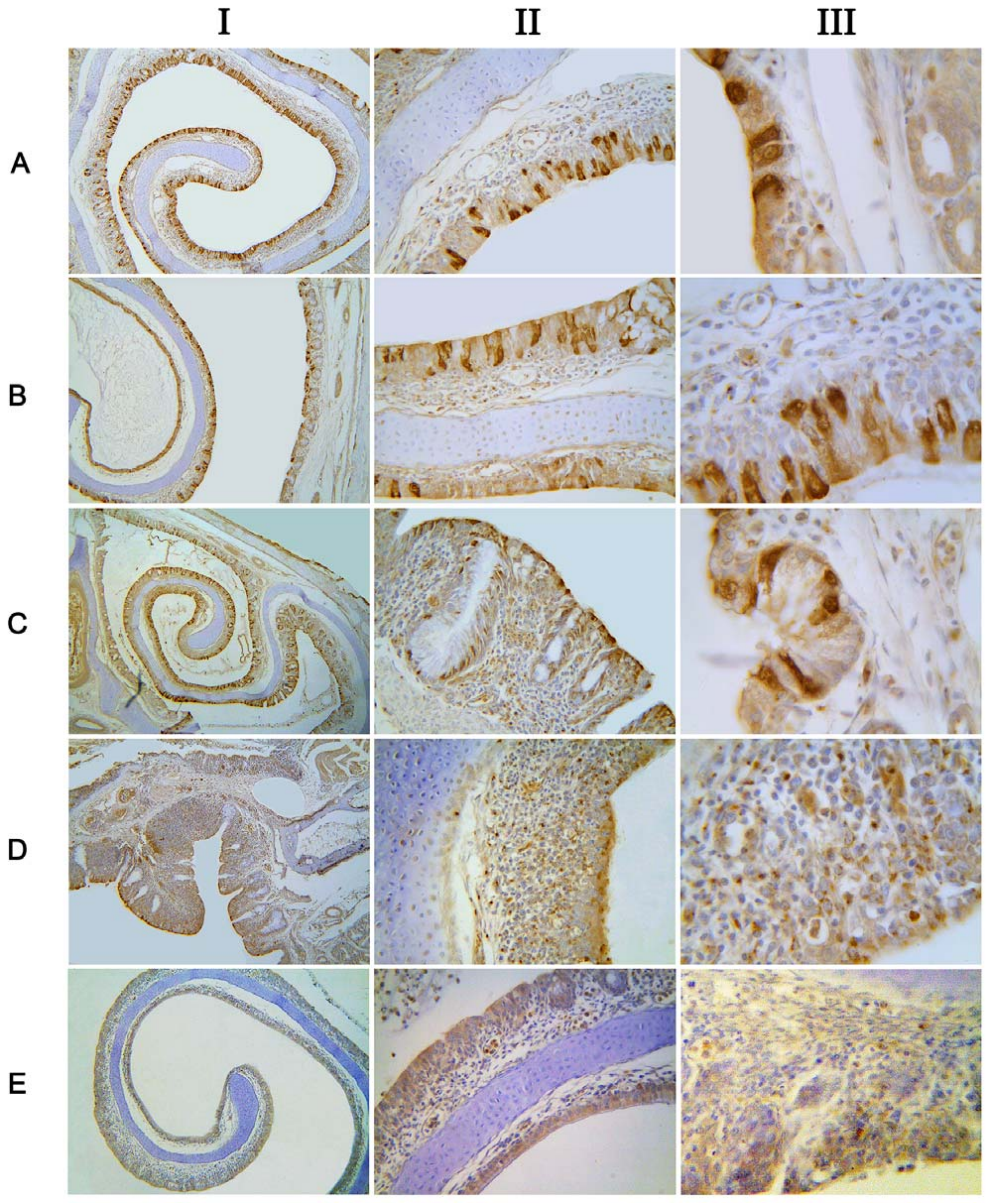
Absorption of OVA by chicken nasal mucosa. (A) Medial side of the concha nasalis media, (B) Lateral side of the concha nasalis media, (C) Mucous gland, (D) NALT, (E) Backgroud control. Column I×100; Column II×200; Column III×400. Essentially similar results were obtained in three independent experiments.

As shown in Figure 5, similar to Trypan Blue, OVA could be extensively absorbed by chicken nasal mucosa except for the stratified squamous epithelium in the front of the nasal cavity. And large differences in uptaking OVA was also observed among the adjacent epithelial cells (Fig. 5A and 5B). The positive cells included ciliated epithelial cell, non-ciliated epithelial cells and goblet cells (Fig. 5C). In contrast to trypan blue, a large number of OVA was absorbed into NLAT and diffused lymphoid tissues, showing dot distribution (Fig. 5D). However, there were fewer OVA in the lamina propria of non-lymphoid tissues (Fig. 5A, 5B and 5C).

### Absorption of FLP

**Figure 6:**
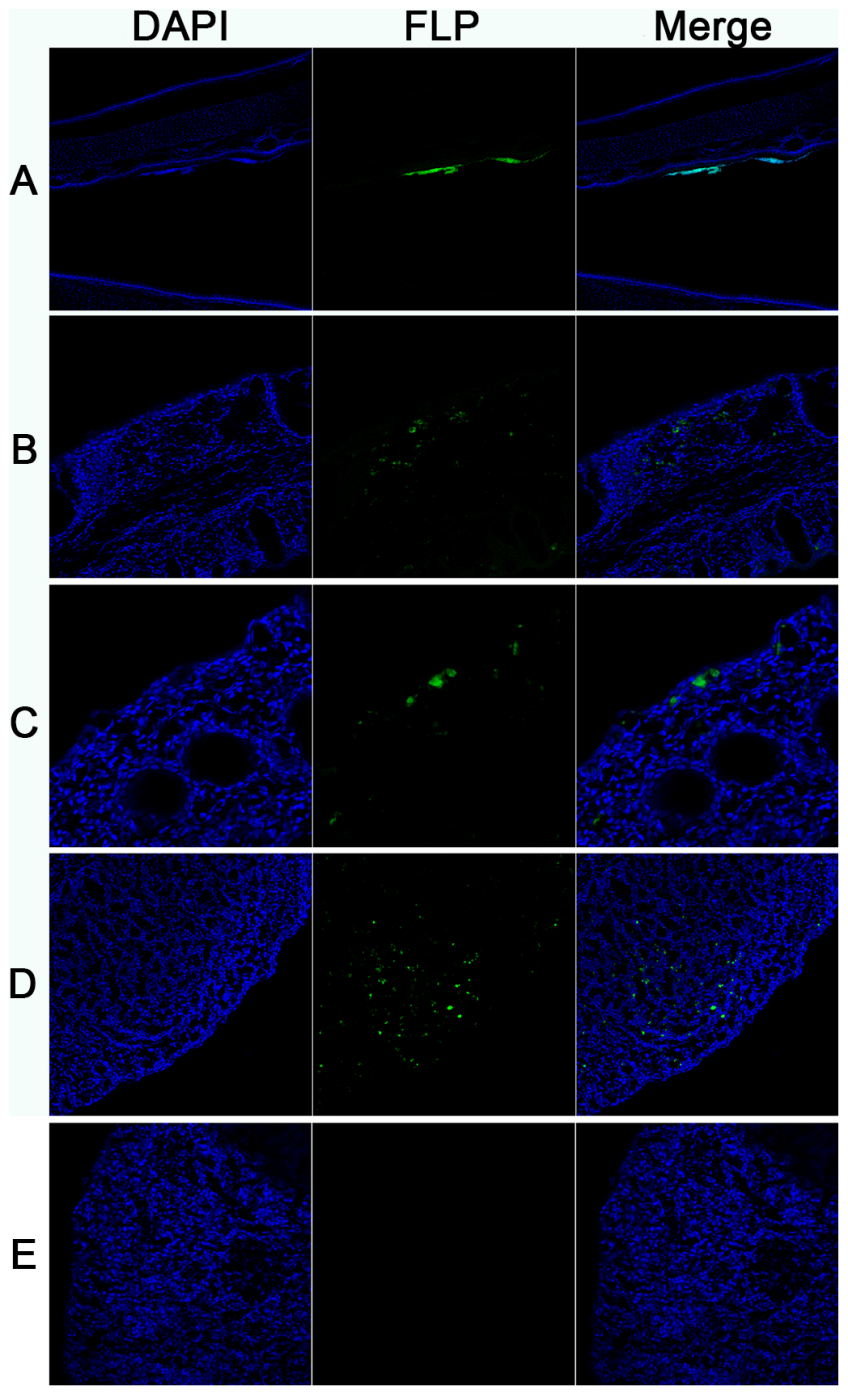
Absorption of 30 nm FLP by chicken nasal mucosa. (A) The forepart of the nasal cavity (×5), (B) Mucosa of the concha nasalis media (×16), (C) Mucous glands (×32), (D) NALTs (×16), (E) Backgroud control. The 30 nm FLP were fluorescent green, the nucleus were staining with DAPI (fluorescent blue). Essentially similar results were obtained in two independent experiments.

**Figure 7:**
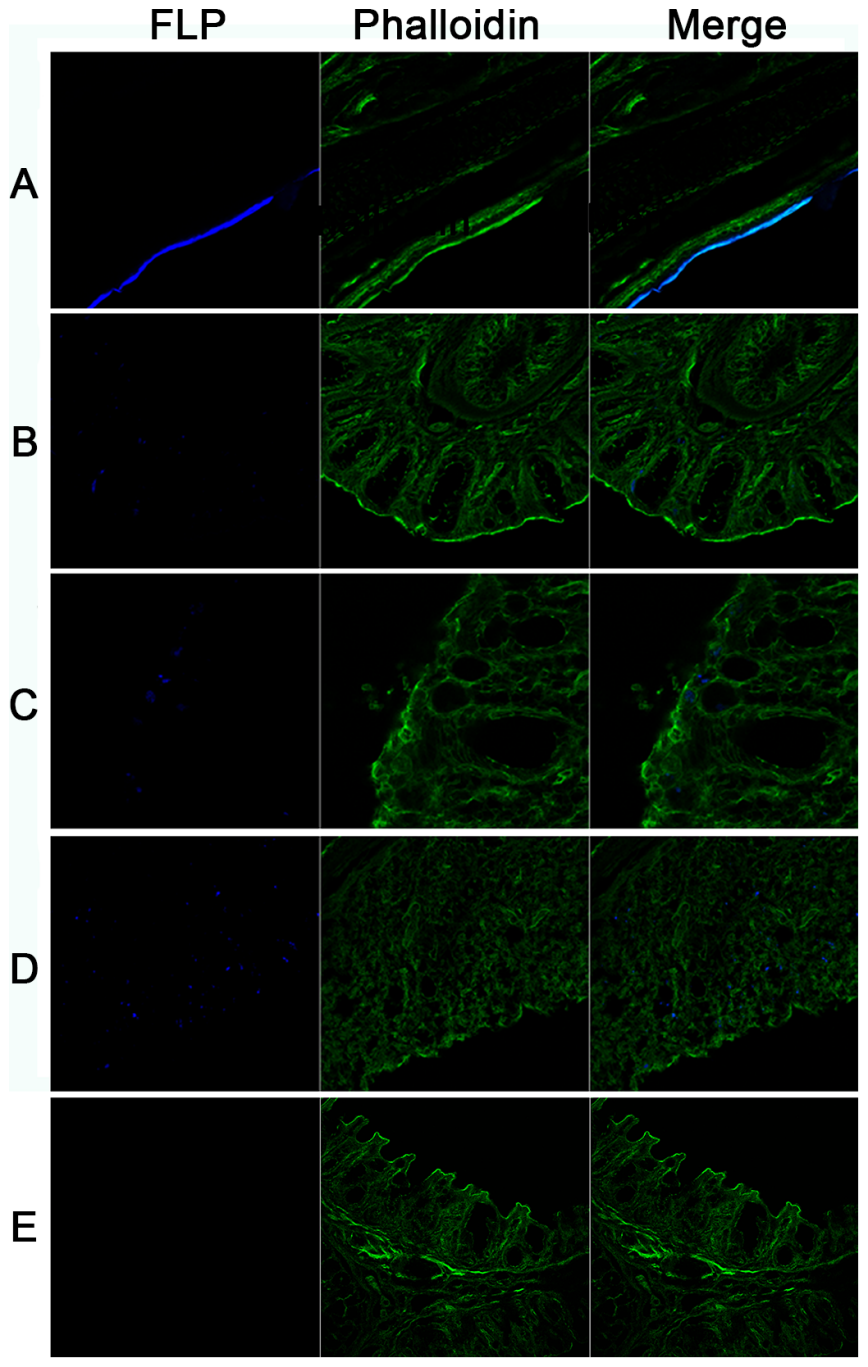
Absorption of 50 nm FLP by chicken nasal mucosa. (A) Stratified squamous epithelium (×5), (B) Mucosa of concha nasalis media (×16), (C) Mucous gland (×32), (D) NALTs (×32), (E) Backgroud control. The 50 nm FLP were fluorescent blue, the microfilament were staining with phalloidin (fluorescent green). Essentially similar results were obtained in two independent experiments.

As shown in Figures 6 and 7, the chicken nasal mucosa had similar absorbing capacity for the 30 nm and 50 nm FLP. FLP could enter the stratified squamous epithelium in the front of the nasal cavity (Fig. 6A, Fig. 7A). Both the FLP could be absorbed by the mucosal epithelium and mucous gland of the nasal cavity (Fig. 6B and 6C, Fig. 7B and 7C), and also could be absorbed and transported through the FAE of NALT (Fig. 6D, Fig. 7D). The results of absorbing position of the 100 nm FLP was similar to the former two in the nasal cavity of chicken (data not shown).

### Absorption of IAIV

**Figure 8:**
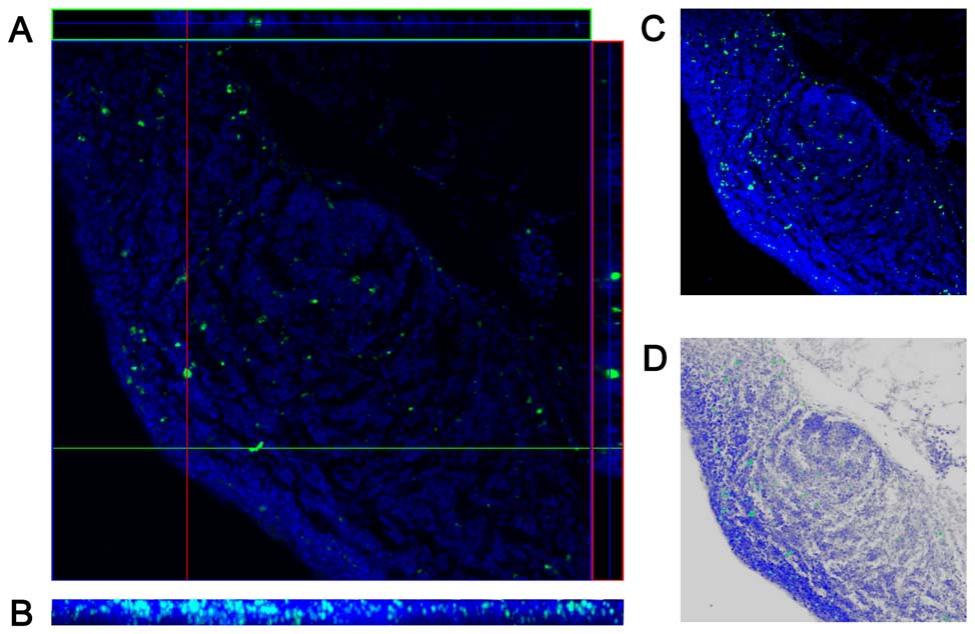
Absorption of IAIV by NALT on the septum. (A) Layer scanning by LSCM (×20), (B) lateral view of three-dimensional image (×20), (C) Merge of layer scanning images (×20), (D) Virtual HE stain of the merged image (×20). Essentially similar results were obtained in two independent experiments.

As similar with 30–100 nm FLP, IAIV was unable to enter the stratified squamous epithelium in the front of nasal cavity, but the pseudostratified ciliated epithelium of the middle nasal cavity. Confocal tomography (Fig. 8A) and synthetic diagram (Fig. 8B and 8C) displayed that IAIV has entered the follicles and inter-follicular area of NALT at 1 h.p.i. The intake of IAIV in NALT was significantly greater than that of other sites, and greater than the 100 nm FLP, which had the similar particle diameter. Virtual staining showed the typical structure of the second lymphoid follicle and the IAIV which had been absorbed in (Fig. 8D).

### Adjuvants enhanced the absorption of IAIV by NALT

**Figure 9:**
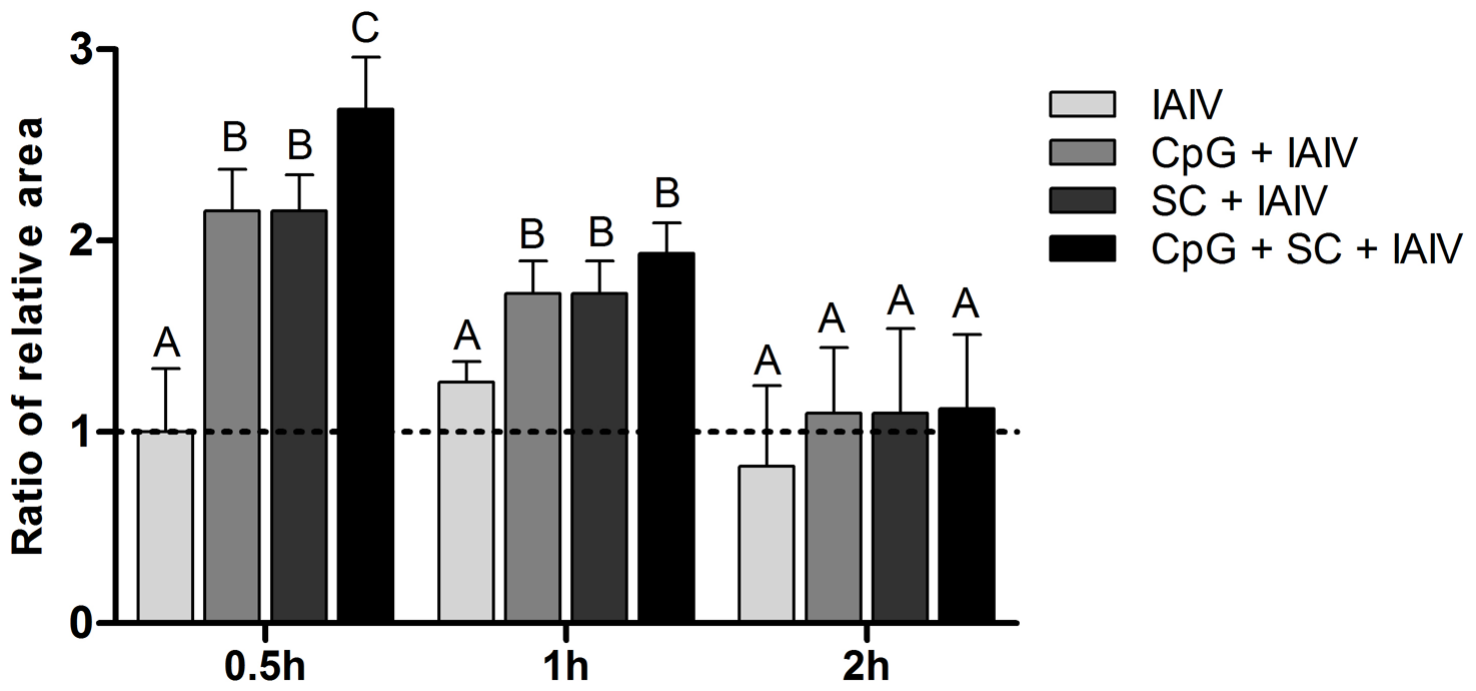
Changes of relative fluorescence area of IAIV in NALT. Significance of difference between groups (*P*<0.01) was calculated using ANOVA. Identical upper-case letters indicate no statistical difference. Values are given as means ± SEM of 2 separate experiments.

To evaluate whether the mucosal adjuvants could promote the absorption of IAIV, and result in immune enhancement, IAIV was intranasal inoculated combined with an absorption enhancer sodium cholate and an immunostimulant CpG. The relative fluorescence area of the single IAIV group at 0.5 h was set as standard, and the ratio of the data from all other groups and time points were shown in Figure 9. The results showed that, compared with single IAIV group, the group of CpG or sodium cholate in combination with IAIV had a significant increase in relative fluorescence area of IAIV in NALT (p<0.01). It meant CpG or sodium cholate effectively improved the intake of IAIV in NALT. The enhancement of the compound adjuvant of CpG and sodium cholate was significantly higher than that of the single adjuvant at 0.5 h.p.i (*P*<0.01). However, there was no significant difference at 2 h.p.i.

## Discussion

Most space of chicken's nasal cavity was occupied by the turbinates. The spiral structures of the turbinates increased the superficial area of the nasal mucosa,which was likely beneficial to prevent the entry of dust and foreign matter. However, the increased mucosal areas also offered more potential absorption sites for the nasal vaccines. Moreover, the existence of NALT and diffuse lymphoid tissues made the nasal mucosa more powerful for antigen recognition and presentation.

NALT has been represented principally in the rodent nasal passage as two separate lymphoid aggregates, which has also been found in cattle, monkeys [19], [20], horses [21], [22] and rabbits [23]. Currently, most studies on NALT were performed on rodents, the favorable animal models for the intranasal delivery and the mechanism of immune response. However, accumulation of lymphoid tissue has been demonstrated in the oculo-nasal region in chickens. Invasive lymphocyte populations consistently found in paranasal organs (nasolacrimal ducts, lateral nasal glands and their ducts) of healthy commercial stock chickens suggested that these tissue systems might be capable of local immune response to environmental stimulation [24]. Besides, in the chicken NALT these large lymphocytes aggregations with GC were covered by non-ciliated squamous epithelium. Moreover, van Alstine and Arp observed that the low cuboidal epithelium covering each lymphoid nodule of the turkey BALT is morphologically similar to the lymphoepithelium of BALT and Peyer's patches (PPs) of chickens and mammals, which lack cilia and mucous-producing cell [25]. On account of these findings, NALT has been deemed to be functionally similar to mucosal lymphoid aggregates in the intestine, such as PPs [26]. As an inductive site of the upper respiratory tract, NALT plays an important role in the local immune activation following the nasal antigen delivery [27]–[29]. Considering lacking of a lymph node system in birds, NALT may serve as the major inductive site for the mucosal immune network [30]. Nonetheless, the absorbing capacity of the respiratory passage is unsatisfactory, resulting from the mucosal barrier and the nasal mucociliary transport process. Hence, the insufficient absorption of antigen by the nasal mucosa may restrict the induction of an effective nasal immune response.

Generally, nasal cavity is considered to cilium the mucosal surface in a short time. The half time of clearance of non-mucoadhesive formulations from the human nasal cavity is about 20 min [31]. Such a rapid clearance time may not allow sufficient retention for antigen to be absorbed through the nasal mucosa [32]. Our study found that the clearance time of chicken nasal cavity was about 15∼40 min, which could be affected by both the liquid dosage and the animal age. Our observations showed that 0.1 ml liquid dropped into the nostrils was capable of filling the entire nasal cavity of a 10-day-old chicken; while for the 60-day-old chicken, the liquid overflowed the ventral wall into the oral cavity through the choanal cleft. In both conditions, the liquid vaccine could reach the NALT located on the ventral wall of the choanal cleft. However, it was difficult for the liquid vaccine to reach the NALT located on the nasal septum of a 60-day-old chicken. In general, it seems that the liquid vaccines are more suitable for chicklings, while the dry powder vaccines are better choices for adult chicken. Furthermore, BALT takes part in bronchial immune processes, unlike PPs, its structure, topography and ability to perform defensive function in birds is largely age-dependent. Fully developed BALT in birds was not observed until 6-week-old, and the number of lymph nodules in birds increased with age [8], [9], [33], [34]. NALT is also acquired developmental mucosa-associated lymphoid tissue. Specifically, there was no lymphoid tissue and lymphocytes aggregation under NALT mucosa of 1-day-old chicks. Lymphocytes aggregation appeared for 7-day-old chicks, but complete lymphatic structure did not form, and the number of lymphocytes and the area of lymphocytes aggregation were small. NALT has basically formed when chicks grew to 14-day-old, the number of lymphocytes increased significantly and secondary lymphoid follicles appeared. Diffuse lymphoid tissues began to randomly distribute under nasal mucosa (e.g. middle turbinate mucosa) except for NALT. For 35-day-old chicken, NALT basically matured. Ripe secondary lymphoid follicles could be watched, and the area and number of follicles increased. Diffuse lymphoid tissues of other parts had also increased area and number. There was no significant difference between 56-day-old and 35-day-old in the morphology and structure of NALT, only 56-day-old chickens had more and greater lymphoid follicles and diffuse lymphoid tissues. Hence, 14-day-old chickens were selected for the evaluation of nasal absorption to different materials.

Trypan blue (MW 960.82 Da) is a vital stain used to selectively color dead tissues or cells blue, and generally considered can not be taken up by the living cells with intact cell membranes. OVA (MW 45 KDa) is the main protein found in egg white, containing 385 amino acids [16]. The dose-dependent effect was observed in the absorption of both trypan blue and OVA by the nasal mucosa, indicated that these two substances might undergo passive transport. However, there was a large difference between the adjacent epithelial cells in absorptive capacity, suggesting that the absorption by the nasal epithelium was correlated with the cell type-specific. Besides, in the posterior part of nasal cavity, trypan blue could enter the cells distributed in the lamina propria and showed dot distribution, suggesting that trypan blue was taken up through the endocytosis or pinocytosis here. Thus, we hypothesized that the uptake of trypan blue in chicken nasal mucosa was likely influenced by both passive transport and active uptake, in contrast to the exclusion by the living cells *in vitro*. Yamamoto et al also found that respiratory mucosa could uptake trypan blue, and the absorption capacity of nasal cavity were greater than that of large intestine and oral cavity [17]. Similar to trypan blue, OVA was mainly taken up by the epithelial cells in the position of non-lymphoid tissues. However, the absorption of OVA by the diffused lymphoid tissue and NALT was significantly stronger than that of trypan blue, and OVA could be transported through the mucosal epithelium to the lymphoid follicles. This might be related with the antigenic characteristics of OVA [35], [36] and the antigen-presenting cells, such as dendritic cells (DC).

FLP is a kind of inactive macromolecule particles, which widely used in the studies of absorption and the identification of microfold cell (M cell) on the FAE [37]–[39]. Our results found that chicken nasal mucosa could intake the 30 nm∼100 nm FLP, while the absorptive capability of 100 nm FLP was weaker than that of 50 nm and 30 nm FLP. A study has also shown that nanoparticles had satisfactory absorption effect in chicken nasal cavity, and antigen-encapsulated immunized better than antigen alone [40]. Interestingly, FLP could be transported through the FAE and reached to the lymphoid follicles of NALT in this study, where there was no trypan blue. Considering that both FLP and trypan blue are inactive particles without antigenicity, and the small molecular trypan blue is likely more easily to be absorbed than FLP. Additionally, non-cliated squamous or cuboidal cells which covered lymphoid nodule showed morphological resemblance to the M-cells which have been demonstrated in the rat NALT [43], [44].Therefore, we speculated that the intake of FLP in NALT was associated with the M cells in FAE. Studies have shown that M cells had the ability of uptaking and transporting large particles across the epithelium [41], [42]. However, Fagerland and Arp found no typical M-cells in the turkey BALT, although epithelial cells are attempted and intimately associated with intraepithelial lymphocytes [33]. Furthermore, our study also did not found the cell surface markers of M cells in chicken nasal cavity, failing to show M cells by immunological methods. Thus, the co-localization of FLP and M cells wasn't achieved, and this hypothesis remained to be demonstrated.

IAIV is 100∼200 nm in diameter, which has a similar size to the 100 nm FLP. To facilitate observation, the dose of IAIV used for intranasal inoculation was far more than that of the routine immunization. Our study found that the nasal mucosa of chicken had more powerful uptake for IAIV than the 100 nm FLP, resulting from the antigenicity of IAIV, and IAIV appeared in the follicles and interfollicular area of NALT at 0.5 h.p.i, indicating that the IAIV could be effective uptaken by NALT and result in the activation of immune response. Hence, IAIV administered through the nasal route may be a potential vaccine for AIV prevention. On the other hand, IAIV could also be taken in the mucosal epithelium of non-lymphoid tissues. Considering the IELs in the nasal mucosa, the diffuse lymphoid tissues distributed under the epithelium of turbinates and the characteristics of highly vascularized, the region of nasal mucosa except for NALT might also be the inductive site of immune response. Researches have demonstrated the mucosal immune response elicited by the epithelial pathway of non-lymphoid tissues [45]–[49], while there was few relevant information in birds. Thus the hypothesis remained to be demonstrated.

Furthermore, the absorption of IAIV by NALT was significantly increased after combining with CpG or sodium cholate. As an absorption enhancer, sodium cholate can open the tight junctions between epithelial cells and promote the intercellular transport through the mucosal epithelium. At the same time, CpG is an immunostimulant, with no direct effects on the absorption. However, the intake of IAIV by NALT was increased significantly after intranasal inoculation with CpG, which might be relevant with the DCs in NALT. Studies have shown that the DCs in PPs could complete the antigen uptake within 15∼30 min. There were also DCs in NALT, and studies have reported that CpG was a strong stimulation to DCs. It is likely that CpG first stimulated the DCs in NALT and enhanced the ability of DCs to capture and to present exogenous antigen, resulting in the increasing intake of IAIV in NALT. It was a pity that our study failed to show the DCs in NALT of chicken nasal cavity through the immunological method. Thus we missed the co-localization of nasal DCs and IAIV.

## Conclusions

In this study, anatomical and histological characteristic of chicken nasal cavity were systematically studied. NALT of chicken was mainly distributed in the root of nasal septum and the dorsal side of choanal cleft, which mainly consisted of second lymphoid follicles. Besides, diffuse lymphoid follicles and IELs were also present in other regions of nasal mucosa. Except the stratified squamous epithelium in the front of nasal cavity, the chicken nasal mucosa had a wide range of absorption for trypan blue, OVA, FLP and IAIV, while the absorptive capacity was different. NALT could effectively uptake IAIV, and the immune adjuvants such as sodium cholate and CpG could improve the intake of IAIV in NALT.

## References

[1] Heritage PL, Underdown BJ, Arsenault AL, Snider DP, McDermott MR (1997) Comparison of murine nasal-associated lymphoid tissue and Peyer's patches. Am J Respir Crit Care Med 156: 1256–1262. 9351630 10.1164/ajrccm.156.4.97-03017

[2] Brandtzaeg P, Pabst R (2004) Let's go mucosal: communication on slippery ground. Trends Immunol 25: 570–577. 15489184 10.1016/j.it.2004.09.005

[3] Hiller AS, Tschernig T, Kleemann WJ, Pabst R (1998) Bronchus-associated lymphoid tissue (BALT) and larynx-associated lymphoid tissue (LALT) are found at different frequencies in children, adolescents and adults. Scand J Immunol 47: 159–162. 9496692 10.1046/j.1365-3083.1998.00276.x

[4] Quinteiro P, Salazar I (2005) Secondary or Accessory Glomerular Layer in Mice During Growth. Anatomia, Histologia, Embryologia 34: 41–41.

[5] Kuper CF, Koornstra PJ, Hameleers DM, Biewenga J, Spit BJ, et al (1992) The role of nasopharyngeal lymphoid tissue. Immunol Today 13: 219–224. 1627250 10.1016/0167-5699(92)90158-4

[6] Ohshima K, Hiramatsu K (2000) Distribution of T-cell subsets and immunoglobulin-containing cells in nasal-associated lymphoid tissue (NALT) of chickens. Histology and Histopathology 15: 713–720. 10963115 10.14670/HH-15.713

[7] Smialek M, Tykalowski B, Stenzel T, Koncicki A (2011) Local immunity of the respiratory mucosal system in chickens and turkeys. Pol J Vet Sci 14: 291–297. 21721419 10.2478/v10181-011-0047-2

[8] Reese S, Dalamani G, Kaspers B (2006) The avian lung-associated immune system: a review. Veterinary Research 37: 311–324. 16611550 10.1051/vetres:2006003

[9] Fagerland JA, Arp LH (1993) Distribution and quantitation of plasma cells, T lymphocyte subsets, and B lymphocytes in bronchus-associated lymphoid tissue of chickens: age-related differences. Reg Immunol 5: 28–36. 8347468

[10] Hiller A, Tschernig T, Kleemann W, Pabst R (1998) Bronchus-associated lymphoid tissue (BALT) and larynx-associated lymphoid tissue (LALT) are found at different frequencies in children, adolescents and adults. Scandinavian journal of immunology 47: 159–162. 9496692 10.1046/j.1365-3083.1998.00276.x

[11] Harkema JR, Carey SA, Wagner JG (2006) The nose revisited: a brief review of the comparative structure, function, and toxicologic pathology of the nasal epithelium. Toxicol Pathol 34: 252–269. 16698724 10.1080/01926230600713475

[12] Xiaowen Z, Qinghua Y, Xiaofei Z, Qian Y (2009) Co-administration of inactivated avian influenza virus with CpG or rIL-2 strongly enhances the local immune response after intranasal immunization in chicken. Vaccine 27: 5628–5632. 19647063 10.1016/j.vaccine.2009.07.023

[13] Yuanji G, Xiaowen C (1997) influenza virus and experimental technology. Beijing: China Three Gorges press 249 p.

[14] Tamura S, Iwasaki T, Thompson AH, Asanuma H, Chen Z, et al (1998) Antibody-forming cells in the nasal-associated lymphoid tissue during primary influenza virus infection. J Gen Virol 79 (Pt 2): 291–299. 9472613 10.1099/0022-1317-79-2-291

[15] Zhang XW, Yu QH, Zhang XF, Yang Q (2009) Co-administration of inactivated avian influenza virus with CpG or rIL-2 strongly enhances the local immune response after intranasal immunization in chicken. Vaccine 27: 5628–5632. 19647063 10.1016/j.vaccine.2009.07.023

[16] Liu G, Song J, Guo Y, Wang T, Zhou Z (2013) Astragalus injection protects cerebral ischemic injury by inhibiting neuronal apoptosis and the expression of JNK3 after cerebral ischemia reperfusion in rats. Behav Brain Funct 9: 36. 24083559 10.1186/1744-9081-9-36 PMC3850702

[17] Louis KS, Siegel AC (2011) Cell viability analysis using trypan blue: manual and automated methods. Mammalian Cell Viability: Springer. pp. 7–12. 10.1007/978-1-61779-108-6_2 21468962

[18] Strober W (2001) Trypan blue exclusion test of cell viability. Curr Protoc Immunol Appendix 3: Appendix 3B. 10.1002/0471142735.ima03bs21 18432654

[19] Loo SK, Chin KN (1974) Lymphoid tissue in the nasal mucosa of primates, with particular reference to intraepithelial lymphocytes. J Anat 117: 249–259. 4461719 PMC1231399

[20] Hameleers DM, van der Ende M, Biewenga J, Sminia T (1989) An immunohistochemical study on the postnatal development of rat nasal-associated lymphoid tissue (NALT). Cell Tissue Res 256: 431–438. 2731226 10.1007/BF00218901

[21] Mair TS, Batten EH, Stokes CR, Bourne FJ (1987) The histological features of the immune system of the equine respiratory tract. J Comp Pathol 97: 575–586. 3680645 10.1016/0021-9975(87)90008-9

[22] Mair TS, Batten EH, Stokes CR, Bourne FJ (1988) The distribution of mucosal lymphoid nodules in the equine respiratory tract. J Comp Pathol 99: 159–168. 3183086 10.1016/0021-9975(88)90069-2

[23] Casteleyn C, Broos AM, Simoens P, Van den Broeck W (2010) NALT (nasal cavity-associated lymphoid tissue) in the rabbit. Vet Immunol Immunopathol 133: 212–218. 19733916 10.1016/j.vetimm.2009.08.011

[24] Bang BG, Bang FB (1968) Localized lymphoid tissues and plasma cells in paraocular and paranasal organ systems in chickens. Am J Pathol 53: 735–751. 5693341 PMC2013518

[25] Arai N, Hashimoto Y, Kitagawa H, Kon Y, Kudo N (1988) Immunohistochemical study on the distribution of lymphoid tissues in the upper alimentary and respiratory tracts of chickens. Nihon Juigaku Zasshi 50: 183–192. 3283394 10.1292/jvms1939.50.183

[26] Harmsen A, Kusser K, Hartson L, Tighe M, Sunshine MJ, et al (2002) Cutting edge: organogenesis of nasal-associated lymphoid tissue (NALT) occurs independently of lymphotoxin-alpha (LT alpha) and retinoic acid receptor-related orphan receptor-gamma, but the organization of NALT is LT alpha dependent. J Immunol 168: 986–990. 11801629 10.4049/jimmunol.168.3.986

[27] Fujimura Y (2000) Evidence of M cells as portals of entry for antigens in the nasopharyngeal lymphoid tissue of humans. Virchows Archiv 436: 560–566. 10917169 10.1007/s004289900177

[28] Sato S, Kiyono H (2012) The mucosal immune system of the respiratory tract. Curr Opin Virol 2: 225–232. 22542216 10.1016/j.coviro.2012.03.009

[29] Zuercher AW, Coffin SE, Thurnheer MC, Fundova P, Cebra JJ (2002) Nasal-associated lymphoid tissue is a mucosal inductive site for virus-specific humoral and cellular immune responses. Journal of Immunology 168: 1796–1803. 10.4049/jimmunol.168.4.1796 11823512

[30] Ridley Lathers DM, Gill RF, Montgomery PC (1998) Inductive pathways leading to rat tear IgA antibody responses. Invest Ophthalmol Vis Sci 39: 1005–1011. 9579480

[31] Illum L (2003) Nasal drug delivery—possibilities, problems and solutions. J Control Release 87: 187–198. 12618035 10.1016/s0168-3659(02)00363-2

[32] Pawar D, Goyal AK, Mangal S, Mishra N, Vaidya B, et al (2010) Evaluation of mucoadhesive PLGA microparticles for nasal immunization. AAPS J 12: 130–137. 20077052 10.1208/s12248-009-9169-1 PMC2844518

[33] Fagerland JA, Arp LH (1990) A morphologic study of bronchus-associated lymphoid tissue in turkeys. Am J Anat 189: 24–34. 2239743 10.1002/aja.1001890104

[34] Fagerland JA, Arp LH (1993) Structure and development of bronchus-associated lymphoid tissue in conventionally reared broiler chickens. Avian Dis 37: 10–18. 8452486

[35] Golias J, Schwarzer M, Wallner M, Kverka M, Kozakova H, et al (2012) Heat-induced structural changes affect OVA-antigen processing and reduce allergic response in mouse model of food allergy. PLoS One 7: e37156. 22629361 10.1371/journal.pone.0037156 PMC3357411

[36] Qian L, Qian GX (2002) [Antigen presentation efficiency of recombinant Vaccinia virus expressing minigene product OVA(257-264) in eukaryotic cells]. Sheng Wu Hua Xue Yu Sheng Wu Wu Li Xue Bao (Shanghai) 34: 719–724. 12417913

[37] Pappo J, Ermak TH (1989) Uptake and translocation of fluorescent latex particles by rabbit Peyer's patch follicle epithelium: a quantitative model for M cell uptake. Clin Exp Immunol 76: 144–148. 2661061 PMC1541725

[38] Brayden DJ (2001) Oral vaccination in man using antigens in particles: current status. Eur J Pharm Sci 14: 183–189. 11576821 10.1016/s0928-0987(01)00175-0

[39] Liu H, Meagher CK, Moore CP, Phillips TE (2005) M cells in the follicle-associated epithelium of the rabbit conjunctiva preferentially bind and translocate latex beads. Invest Ophthalmol Vis Sci 46: 4217–4223. 16249501 10.1167/iovs.05-0280

[40] Zhao K, Chen G, Shi XM, Gao TT, Li W, et al (2012) Preparation and efficacy of a live newcastle disease virus vaccine encapsulated in chitosan nanoparticles. PLoS One 7: e53314. 23285276 10.1371/journal.pone.0053314 PMC3532065

[41] Mabbott NA, Donaldson DS, Ohno H, Williams IR, Mahajan A (2013) Microfold (M) cells: important immunosurveillance posts in the intestinal epithelium. Mucosal Immunol 6: 666–677. 23695511 10.1038/mi.2013.30 PMC3686595

[42] Kanaya T, Hase K, Takahashi D, Fukuda S, Hoshino K, et al (2012) The Ets transcription factor Spi-B is essential for the differentiation of intestinal microfold cells. Nat Immunol 13: 729–736. 22706340 10.1038/ni.2352 PMC3704196

[43] Frieke Kuper C, Koornstra PJ, Hameleers DM, Biewenga J, Spit BJ, et al (1992) The role of nasopharyngeal lymphoid tissue. Immunology today 13: 219–224. 1627250 10.1016/0167-5699(92)90158-4

[44] Spit BJ, Hendriksen EG, Bruijntjes JP, Kuper CF (1989) Nasal lymphoid tissue in the rat. Cell Tissue Res 255: 193–198. 2736604 10.1007/BF00229081

[45] Maldonado-Contreras AL, McCormick BA (2011) Intestinal epithelial cells and their role in innate mucosal immunity. Cell Tissue Res 343: 5–12. 21104188 10.1007/s00441-010-1082-5 PMC4131254

[46] Nasu K, Itoh H, Yuge A, Nishida M, Narahara H (2007) Human oviductal epithelial cells express Toll-like receptor 3 and respond to double-stranded RNA: Fallopian tube-specific mucosal immunity against viral infection. Hum Reprod 22: 356–361. 17043099 10.1093/humrep/del385

[47] Itoh H, Nasu K, Nishida M, Matsumoto H, Yuge A, et al (2006) Human oviductal stromal fibroblasts, but not oviductal epithelial cells, express Toll-like receptor 4: the site-specific mucosal immunity of the human fallopian tube against bacterial infection. Am J Reprod Immunol 56: 91–101. 16836611 10.1111/j.1600-0897.2006.00389.x

[48] Lee HJ, Choi SC, Choi EY, Lee MH, Seo GS, et al (2005) Iron chelator induces MIP-alpha/CCL20 in human intestinal epithelial cells: implication for triggering mucosal adaptive immunity. Exp Mol Med 37: 297–310. 16155407 10.1038/emm.2005.40

[49] Tlaskalova-Hogenova H, Farre-Castany MA, Stepankova R, Kozakova H, Tuckova L, et al (1995) The gut as a lymphoepithelial organ: the role of intestinal epithelial cells in mucosal immunity. Folia Microbiol (Praha) 40: 385–391. 8763152 10.1007/BF02814746

